# Difficulties in access and estimates of public beds in intensive care units in the state of Rio de Janeiro

**DOI:** 10.1590/S1518-8787.2016050005997

**Published:** 2016-04-27

**Authors:** Rosane Sonia Goldwasser, Maria Stella de Castro Lobo, Edilson Fernandes de Arruda, Simone Aldrey Angelo, José Roberto Lapa e Silva, André Assis de Salles, Cid Marcos David

**Affiliations:** I Divisão Médica. Hospital Universitário Clementino Fraga Filho. Universidade Federal do Rio de Janeiro. Rio de Janeiro, RJ, Brasil; II Serviço de Epidemiologia e Avaliação. Hospital Universitário Clementino Fraga Filho. Universidade Federal do Rio de Janeiro. Rio de Janeiro, RJ, Brasil; III Programa de Pós-Graduação de Engenharia de Produção. Instituto Alberto Luiz Coimbra de Pós-Graduação e Pesquisa de Engenharia. Universidade Federal do Rio de Janeiro. Rio de Janeiro, RJ, Brasil; IV Departamento de Clínica Médica. Faculdade de Medicina. Universidade Federal do Rio de Janeiro. Rio de Janeiro, RJ, Brasil; V Departamento de Engenharia Industrial. Escola Politécnica. Universidade Federal do Rio de Janeiro. Rio de Janeiro, RJ, Brasil

**Keywords:** Intensive Care Units, supply & distribution, Hospital Bed Capacity, Length of Stay, Health Services Accessibility, Equity in Access, Unified Health System, Time Series Studies, Systems Theory

## Abstract

**OBJECTIVE:**

To estimate the required number of public beds for adults in intensive care units in the state of Rio de Janeiro to meet the existing demand and compare results with recommendations by the Brazilian Ministry of Health.

**METHODS:**

The study uses a hybrid model combining time series and queuing theory to predict the demand and estimate the number of required beds. Four patient flow scenarios were considered according to bed requests, percentage of abandonments and average length of stay in intensive care unit beds. The results were plotted against Ministry of Health parameters. Data were obtained from the State Regulation Center from 2010 to 2011.

**RESULTS:**

There were 33,101 medical requests for 268 regulated intensive care unit beds in Rio de Janeiro. With an average length of stay in regulated ICUs of 11.3 days, there would be a need for 595 active beds to ensure system stability and 628 beds to ensure a maximum waiting time of six hours. Deducting current abandonment rates due to clinical improvement (25.8%), these figures fall to 441 and 417. With an average length of stay of 6.5 days, the number of required beds would be 342 and 366, respectively; deducting abandonment rates, 254 and 275. The Brazilian Ministry of Health establishes a parameter of 118 to 353 beds. Although the number of regulated beds is within the recommended range, an increase in beds of 122.0% is required to guarantee system stability and of 134.0% for a maximum waiting time of six hours.

**CONCLUSIONS:**

Adequate bed estimation must consider reasons for limited timely access and patient flow management in a scenario that associates prioritization of requests with the lowest average length of stay.

## RESUMO

**OBJETIVO:**

Determinar o número necessário de leitos públicos de unidades de terapia intensiva para adultos no estado do Rio de Janeiro para atender à demanda existente, e comparar os resultados com a recomendação do Ministério da Saúde.

**MÉTODOS:**

Seguiu-se modelo híbrido que agrega séries temporais e teoria de filas para prever a demanda e estimar o número de leitos necessários. Foram considerados quatro cenários de fluxo de pacientes, de acordo com as solicitações de vagas, proporção de desistências e tempo médio de permanência no leito de unidade de terapia intensiva. Os resultados foram confrontados com os parâmetros do Ministério da Saúde. Os dados foram obtidos da Central Estadual de Regulação, de 2010 a 2011.

**RESULTADOS:**

Houve 33.101 solicitações médicas para 268 leitos de unidade de terapia intensiva regulados no Rio de Janeiro. Com tempo médio de permanência das unidades de terapia intensiva reguladas de 11,3 dias, haveria necessidade de 595 leitos ativos para garantir a estabilidade do sistema e 628 leitos para o tempo máximo na fila de seis horas. Deduzidas as atuais taxas de desistência por melhora clínica (25,8%), estes números caem para 441 e 471. Com tempo médio de permanência de 6,5 dias, o número necessário seria de 342 e 366 leitos, respectivamente; deduzidas as taxas de desistência, de 254 e 275. O Ministério da Saúde estabelece parâmetro de 118 a 353 leitos. Embora o número de leitos regulados esteja na faixa recomendada, necessita-se incremento de 122,0% de leitos para garantir a estabilidade do sistema e de 134,0% para um tempo máximo de espera de seis horas.

**CONCLUSÕES:**

O dimensionamento adequado de leitos deve considerar os motivos de limitações de acesso oportuno e a gestão do fluxo de pacientes em um cenário que associa priorização das solicitações com menor tempo médio de permanência.

## INTRODUCTION

The universalization of health services implemented by the Brazilian Unified Health System (SUS) has shown poor results in terms of rationalizing action and providing the equitable inclusion of the entire population in public health care, particularly in highly complex and costly activities such as access to intensive care unit (ICU) beds^9^. When the demand for services exceeds their supply, access becomes restricted, patient care is postponed and long waiting queues are formed^13^. Given that ICU plays a decisive role in patient survival, delayed access to ICU beds impacts negatively on clinical results and mortality^4^ ^,^ ^25^.

A few strategies have been created to increase access conditions, such as organizing the State Regulation Centers’ (CER) network, with the aim of improving citizens’ orderly admission to high and medium complexity services and procedures, by theme areas, thus supporting their right to health care. The CERs manage existing health demands and available resources to offer the best care response possible within an opportune time frame^10^. Regulation of ICU beds is based on technical priority criteria, following hierarchical protocols built on consensus by medical specialty societies, giving priority to cases of greater severity^4^ ^,^ ^10^. When there are beds available, the patients’ queue is reduced with the mediation of the regulatory system, enabling the community to control the use of public health beds. In the state of Rio de Janeiro, the CER has regulated ICU public beds since 2005. However, it does not regulate private beds contracted by the SUS.

Demand for ICU beds has grown substantially due to increasing longevity and morbidity among the global population. It is estimated that 60.0% of ICU beds are occupied by patients over 65 years of age and the average length of stay (ALOS) of this age group is seven times greater compared with the younger population^1^. In addition to the higher biological risk of the older population, cultural factors have enhanced the trend for institutionalized death and life-prolonging intervention, without necessarily guaranteeing quality, comfort or a lower death rate. More than 70.0% of deaths currently occur in hospitals and, more specifically, in ICU^2^ ^,^ ^16^.

Regarding supply, there is a trend to reduce the number of general hospital inpatient beds and promote other forms of care such as partial hospitalization services and outpatient treatment, also offered by SUS^12^. The lack of general hospital beds results in a bottleneck in ICU exit and the delayed transference of discharged patients.

The imbalance between supply and demand is also influenced by the fact that ICU patients have highly variable lengths of stay and rarely schedule appointments, which typifies an event with random characteristics. In practical terms, the availability of and need for ICU beds changes dynamically, hindering the adequate planning of necessary resources.

Operational research combines methodologies from various fields of knowledge to support problem structuring, analysis and decision making, using a set of models and practices. Given the randomness of demand, considered by time series, it is possible to use queuing theory to estimate services and plan access to them^4^ ^,^ ^15^ ^,^ ^24^.

The objective of this study was to estimate the necessary number of public beds for adults in intensive care units in the state of Rio de Janeiro to meet current demands and compare the results with recommendations by the Brazilian Ministry of Health.

## METHODS

**Figure 1:**
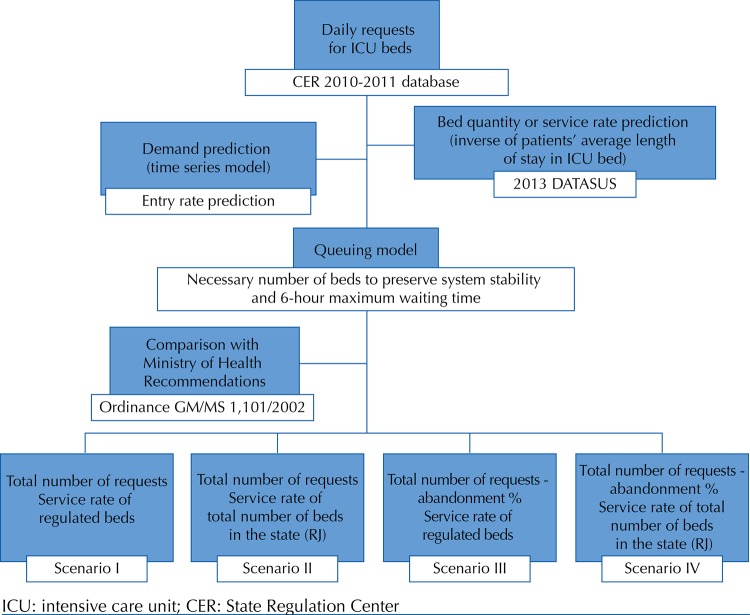
Modeling methodology steps: from demand prediction (based on time series) to number of necessary beds (based on queuing theory) to comparison with Ministry of Health recommendations.

The study investigated a retrospective cohort from the database of the CER of the state of Rio de Janeiro comprising all adult patients who had made requests for daily admission to the 268 ICU beds regulated by the center between 2010 and 2011. These requests enabled the researchers to make future predictions of demand using time series. The number of beds necessary to meet this demand was estimated by queuing theory, considering four different patient flow scenarios. These flow settings considered queue entries (medical requests) and exits, based on the ALOS found in the DATASUS, using the year 2013 as a parameter. The findings were compared with a recommendation by the Brazilian Ministry of Health establishing the number of ICU beds according to population structure. The methodology is synthesized in Figure 1. Time series is a set of chronologically ordered observations used to predict future values based on a series of known data^5^. In this study, CER data and the daily demand per ICU from 2010 to 2011 were organized chronologically to constitute a time series and enable the prediction of future demand.

To predict the future demand for ICU beds, the cohort data were divided into partials of 70.0% and 30.0%, with the first group used for training and the second for validation. The predictions generated by the first subgroup were compared with the actual data of the second. As there are various prediction techniques, such as SARIMA, Holt-Winters and Damped Multiplicative Trend, they were tested and the one with the best performance in predictive potential was chosen – in this case, Damped Multiplicative Trend. The statistic software program R was used to predict expected bed demand for the following year. This prediction was considered for the entry of the queuing model.

Queuing theory is a branch of operational research that studies the formation of queues by their measurable properties^19^. It provides models to study the behavior of a system with random demand growth, allowing it to be sized to satisfy users and be economically feasible for the service provider, avoiding waste of resources and bottlenecks.

The queuing system is characterized by five basic components, namely: arrival process, service time distribution, number of servers, service capacity (maximum number of users supported by the system, in service and in waiting) and queue discipline. In this study, user arrival was measured by time interval between medical requests, in minutes. Service time was measured according to ALOS, in days. For the number of servers, a single queue was considered, directed towards 268 beds, and its capacity was estimated by the minimum number of beds needed to ensure system stability. Finally, queue discipline was organized by order of arrival, based on priority, emulating the CER distribution model for ICU beds.

Given that the observed probability of ICU bed request rates followed the Poisson distribution and ALOS was also exponential, the M/M/s *(*Memoryless/Memoryless/server*)* model was chosen. In this model, arrival intervals and service times have an exponential distribution, independently and identically distributed, and *s* represents the number of service units, in this case the number of ICU beds available for adults. The model is based on the interaction between two parameters: a) mean queue arrival rate (λ = patient/minute), representative of demand; b) mean service rate (μ) or exit rate (1/μ = inverse of ALOS, I days), representative of bed supply. To guarantee system stability, the total service rate, considering all beds in the system, must exceed the demand rate (λ < *s* μ), otherwise the system becomes unstable and the queue tends to infinity.

Once the queue system entry (λ) and exit (μ) parameters have been defined, it is possible to determine, for different scenarios, the minimum number of beds, maximum waiting times and probability curves. For the analyses, a six-hour waiting time is considered feasible to sustain an unchanged prediction, as long as there are appropriate pre-hospital and emergency support measures^4^. Four scenarios were analyzed.

In the current scenario (Scenario I), the calculated arrival rate is based on the total number of requests made to the CER in the state of Rio de Janeiro and the service time parameter is the current ALOS of the 268 ICU beds regulated by the CER.

In the scenario with reduced service time (Scenario II), the arrival rate is still calculated by the total number of requests made to the CER, but the ALOS parameter is reduced, equal to that of all 1,187 ICU beds in the state of Rio de Janeiro (including private beds and those not regulated by the CER).

In Scenario III, abandonments due to clinical improvement are deducted from the arrival rate and the service time parameter is the current ALOS of ICU beds regulated by the CER.

Finally, in Scenario IV, abandonments due to clinical improvement were excluded from the arrival rate and the service time parameter is the reduced ALOS of the total number of ICU beds in the state of Rio de Janeiro.

The results obtained were compared with the recommendation by the Brazilian Ministry of Health issued by Minister’s Office Ordinance 1,101/2002, which provides for care coverage parameters within the SUS scope. The Ordinance establishes the number of ICU beds according to the total population, namely: the ideal number of ICU beds (adults, children, neonates) must vary between 4.0% and 10.0% of the total number of hospital beds, which in turn must be 2.5-3 beds/1,000 inhabitants. That is, the recommended total number of ICU beds must vary between 0.1-0.3/1,000 inhabitants. As 49.5% of ICU beds are destined for adults, the number of ICU beds for adults must vary between 0.05-0.15 beds/1,000 inhabitants. The reference population for the study considered the Rio de Janeiro state population (16,447,129 inhabitants)^a^, the percentage of inhabitants strictly dependent on SUS (63.4% with no private health insurance)^b^ and the percentage of regulated beds among the total number of ICU beds for adults in the state (22.6%), totaling 2,354,309 individuals.

## RESULTS

Over the period covered by the study, there were 33,101 medical requests for the 268 ICU beds regulated by the CER. During that period, 55.0% of the individuals abandoned the queue and 20.0% died before admission. Among the causes for abandonment, 47.0% were due to clinical improvement or recovery, 35.0% obtained their own transfer and 9.0% were diagnosed outside the regulation profile; the remaining causes were classified as: lack of contact with the requesting unit (3.0%), discharge against medical advice (3.0%), family refusal (2.0%), no means of transportation (1.0%) and indication for outpatient treatment (1.0%). The Table features arrival rate, service time, exit rate and number of ICU beds predicted as necessary to preserve system stability and for a maximum waiting time if six hours in each studied scenario.

**Table:** Parameters and results obtained for each studied scenario. Rio de Janeiro, 2010-2011.

Scenarios	I (current)	II	III	IV
Arrival rate (λ = patients/hour)	2.198204	2.198204	1.635614	1.635614
ALOS (days)	11.3	6.5	11.3	6.5
Exit rate (µ = patients/hour)	0.003702	0.006442	0.003702	0.006442
Number of necessary beds to ensure system stability	595	342	441	254
Number of ICU beds for a maximum waiting time of six hours, with 95.0% of probability	628	366	471	275

ALOS: average length of stay; ICU: intensive care unit

In scenarios I and II, the number of daily requests for ICU beds was 52.76 and the mean interval between requests was 0.46 hours. The demand predicted for a year generated a λ entry rate of 2.198204 patients/hour. In scenarios III and IV, the average arrival rate (minus abandonments due to clinical improvement) was λ = 2.198204 x 0.742 = 1.635614 patients/hour. In scenarios I and III, the ALOS in units regulated by CER was 11.25 days, therefore, µ = 0.003702 patients/hour. In scenarios II and IV, the average length of stay in beds in all hospitals in the state of Rio de Janeiro (public and private) was 6.47 days, therefore, μ = 0.006442 patients/hour.

The number of ICU beds for adults necessary to preserve system stability would be 595 in the current scenario (I), 342 in scenario II, 441 in scenario III and 254 in scenario IV. To ensure a maximum waiting time of six hours for 95.0% of patients, the demand for ICU beds for adults increases to 628, 366, 471 and 275, respectively.

**Figure 2:**
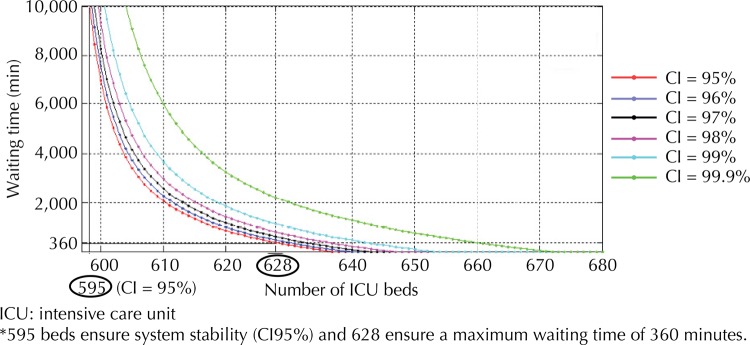
ICU beds and maximum waiting time with a 95% to 99% confidence interval – Scenario I.

Figure 2 shows, for the current scenario (I), the minimum number of beds, maximum waiting times and probability curves corresponding to the confidence intervals of 95.0% to 99.9%. It is possible to graphically relate bed quantity and respective waiting time, according to the confidence interval adopted, by drawing straight lines from bed quantity and waiting time, with the intersection in the confidence interval (CI) curve.

In Figure 2, for the current scenario, with a 95% confidence interval, 595 beds would guarantee stability and 628 would ensure a maximum waiting time of six hours. Maintaining the current patient entry and exit parameters, it would be necessary to increase the number of beds by 122.0% for the queue not to grow indefinitely and by 130.0% to achieve a maximum six-hour waiting time. Inasmuch as an increase in waiting time is accepted, it is possible to work with fewer beds, but always above the minimum stability value. These same curves can be developed for the different scenarios.

**Figure 3:**
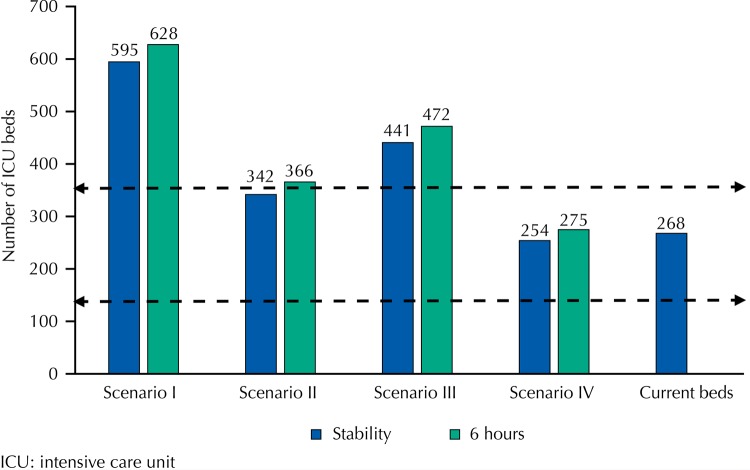
Number of beds prescribed by the Brazilian Ministry of Health, according to current scenario and quantity.

According to the parameters recommended by the Ministry of Health Ordinance, the total number of critical beds in the state of Rio de Janeiro should be between 235 and 705. Considering only ICU beds for adults, the figures vary between 118 and 353. Figure 3 shows the necessary number of beds for each studied scenario and the number of beds currently regulated by the CER and compares them with the limits defined by current legislation. The number of beds currently regulated by the CER, the stability value for scenario II and both values for scenario IV are within the range recommended by the Ministry of Health for the study’s reference population.

## DISCUSSION

Health care managers often underestimate the number of necessary ICU beds, since they do not consider the stochastic character of patient flow^14^. This study proposed a hybrid approach based on time series and queuing theory to make a prediction concerning those beds. The resulting figures are compatible with those of the Brazilian Ministry of Health only for specific demand and length of stay parameters. McManus et al.^14^ showed that, in a randomized model, as in ICUs, queuing theory enables a more appropriate prediction of bed quantity. The proposed approach is, therefore, a useful tool for the planning and operationalization of regulation centers or other bodies managing patient flow.

The current consolidation process of the Brazilian Unified Health System (SUS) prioritizes the organization of health care networks and the expansion of citizens’ access to health services at various levels of need. One of the normative mechanisms for health care inclusion is the regulatory complex, responsible for organizing, controlling, managing and prioritizing access and patient flows, providing appropriate alternatives to citizens’ needs, such as ICU beds^10^.

Occupation of ICU beds and its impact on patient flow has been a great problem in the health sector. Health service flow depends on the quantity of services available (ICU beds), on the demand for this service (entry), on freeing up beds (exit) and prioritizing decision-making, besides other technical, ethical, legal and social criteria that influence access regulation. Imbalance among these factors leads to waiting queues^7^.

In addition to the actual estimate, the analysis of the scenarios suggested that some management aspects might influence bed regulation. The analysis covered from the current scenario (I), in which the patient flow requires twice as many beds as offered by the CER to maintain queue balance, to a scenario in which the necessary values totally comply with Ministry of Health requirements. In other words, in a higher performance scenario, characterized by a shorter length of stay in ICU beds, associated to prioritization of indications (Scenario IV), the number of beds currently available would meet the demand, both in preserving system stability and ensuring a maximum waiting time of six hours, besides complying with current Brazilian regulations.

In terms of bed management, a lower ALOS can reduce the need for beds. Thus Scenario II indicated an improved performance of the system when the average length of stay in ICU beds was reduced by 58.0%. With this simple measure, the need for ICU beds to stabilize the queue and ensure a maximum waiting time of six hours would fall by approximately 40.0%. Adequate use of resources is a priority in health management and ALOS, an important indicator of ICU efficiency, can be used to control costs and compare hospitals. Silva et al.^23^ showed that delayed discharge is mainly associated to processes (such as waiting for procedures and interconsultations) that can be improved with low cost measures, such as intervention by the care team and management^23^. In addition, to represent care quality, it is important to associate ALOS with severity-adjusted mortality rates, since a short length of stay may also be associated to the death of inpatients^11^ ^,^ ^22^. Studies have shown that the ALOS tends to be longer in public than in private hospitals, despite similar mortality indicators, which might be associated to a selection bias^9^ ^,^ ^17^. Nevertheless, it is necessary to investigate the reasons behind the longer ALOS encountered in CER regulated hospitals.

Despite not being within the scope of this study, it is worth investigating the reason for the significant percentage of patients abandoning the queue due to clinical improvement, whether efficiency of pre-hospital care or inappropriate a priori indication criteria. Adequate care in pre-hospital environments, following care protocols, might foster clinical and diagnostic improvement, avoiding indications for ICU admission. In addition, correct and advanced intervention in patients with severe sepsis or acute coronary syndrome, before ICU admission, reduces mortality^3^ ^,^ ^18^ ^,^ ^20^ ^,^ ^25^.

On the other hand, it is important to investigate among the 20.0% of deaths in the queue how many could have been prevented with a shorter waiting time or how many patients arrived at the emergency services beyond therapeutic care.

Resizing requests by 26.0% reduced the need for new ICU beds, due to the lower queue entry rate (Scenario III), simulating a situation of redefined indications and priorities^7^. The key elements of the principles governing priorities in decision making by regulation and protocols involve transparency, rationality and ensuring that these procedures are actually carried out^10^.

In Brazil, decision making regarding indication for ICU admission and discharge lies strictly with the doctor. In the studied cohort, 9.0% of indications were outside the regulation profile and 1.0% would be referred to outpatient treatment. In this context, developing protocols ensures improved risk classification and prioritization of critical cases. In addition, 34.0% of abandonments occurred due to private transfer, which indicates low regulation resoluteness and queue delay. A study of waiting lists in Spain showed that timely access, using queue entry management with priority levels, has a greater impact than increasing bed supply^6^.

Regarding the Brazilian legislation, rules for planning hospital beds and services are traditionally based on historical data series. The fixed variation range for bed quantity does not consider managerial adjustments related to queue entry and exit, such as pre-hospital care, triage of ICU admission indications and ALOS, which have a high influence on the need for such beds^8^ ^,^ ^9^. Demand for hospital beds also varies according to factors such as demographic profile and morbidity burden which, in turn, are subject to change over time. In this sense, it is important to develop methodologies to include such components^21^.

A limitation of this study is the fact that only CER regulated ICU beds were considered in the analysis. Moreover, no distinction was made between different groups of diseases, such as cardiovascular, neurosurgical, trauma, which might have different impacts on factors that influence queue entry and exit, or require regulation for specialized beds, with specific human and technological resources. In this case, the model would have considered more than one waiting list.

Finally, the growing demand for intensive care beds with no corresponding growth in the offer of services increases the challenges of meeting the SUS prescriptions of equity and universal access to health actions and services. To close the gap between demand and supply of ICU beds, adequate bed sizing and system stability must consider reasons for limited access, waiting times and patient flow management, establishing explicit protocols and priorities for entry and quality management of bed occupancy.

## Footnotes

a Instituto Brasileiro de Geografia e Estatística. Estimativas populacionais para os municípios brasileiros em 1.7.2013. Rio de Janeiro (RJ); 2013 [cited 2015 Sept 10]. Available from: http://www.ibge.gov.br/home/estatistica/populacao/estimativa2013/

b Agência Nacional de Saúde Complementar. Taxa de cobertura de planos de saúde - Brasil. Rio de Janeiro; s.d. [cited 2015 Sept 10]. Available from: http://www.ans.gov.br/anstabnet/cgi-bin/dh?dados/tabnet_tx.def
